# Supplementary material for: Anaesthetists’ attitudes towards attending the funerals of their patients: A cross-sectional study among Australian and New Zealand anaesthetists
Source: PLoS One. 2020 Nov 5;15(11):e0239996. doi: 10.1371/journal.pone.0239996 (PMC7643987; doi:10.1371/journal.pone.0239996)
Supplement: S1 Appendix — (PDF) [file pone.0239996.s001.pdf]

1. For patients to whom you have provided anaesthesia care, have you ever attended their funeral?

- o Yes
- o No

2. For patients to whom you have provided anaesthesia care, how often do you form a special bond with the patients or their families?

- o Usually
- o Often
- o Seldom
- o Never

3. Do you think having a special bond with patients or their families makes you more likely to attend the funeral of the patient?

- o Likert scale (Strongly agree, Agree, Neutral, Disagree, Strongly disagree)

4. Do you think you are more likely to attend the funeral of a patient who died unexpectedly compared to someone who died expectedly?

- o Likert scale (Strongly agree, Agree, Neutral, Disagree, Strongly disagree)

5. What do you think are benefits for the ANAESTHETIST who attends a patient's funeral? **(Multiple options can be chosen).** If there are no benefits, please do not tick any box.

- o Gain a greater understanding of whom the patient was before the illness
- o Establish your own professional development
- o Appear professional to the family
- o Pay a gesture of respect to the deceased or their families
- o Express personal grief at the loss of someone you cared
- o Provide comfort and ongoing care for the bereaved family

6. Are there any other benefits to **YOU** by attending the funeral?

- o Textbox where participants can provide a written answer(s)

7. What do you think are the benefits to the **FAMILY** when you attend the funeral of a patient you provided anaesthesia care for? **(Multiple options can be chosen).** If there are no benefits, please do not tick any box.

- o Pay a gesture of respect to the family
- o Provide an opportunity for family members to ask unanswered questions
- o Reduce the family’s stress
- o Extend the relationship to relatives
- o Show caring for patients at the end-of-life and beyond

8. Are there any other benefits to the **FAMILY** by you attending the funeral?

- o Textbox where participants can provide a written answer(s)

9. What are the **BARRIERS** to attending the funeral of a patient you provided anaesthesia care? **(Multiple options can be chosen).** If there are no barriers, please do not tick any box.

- o Time restraint
- o Funeral attendance is unprofessional
- o Funeral attendance is a source of emotional stress for me
- o Personal bereavement from the loss of the patient
- o Presence of the anaesthetist can be traumatic to the family
- o Attending can invite inappropriate questions
- o Attending can invite recriminations and even anger
- o It can disturb the very personal and private grieving process of a family
- o May have implications for anaesthetist-patient confidentiality
- o May be misinterpreted or seen as not warranted
- o Perceived patient and/or family dissatisfaction with care

10. Are there other barriers to attending the funeral?

- o Textbox where participants can provide a written answer(s)

11. What is your age?

- o Younger than 30
- o 30 to 39

- 40 to 49
- 50 to 59
- 60 or older

12. What is your gender?

- Male
- Female

13. How long have you been a consultant anaesthetist for?

- Less than 5 years
- 5 to 10 years
- 10 to 20 years
- More than 20 years

14. Which state of Australia is your practice based on? If you practice in New Zealand please tick the last option

- NSW
- QLD
- ACT
- VIC
- SA
- WA
- NT
- TAS
- New Zealand

15. Do you work in an urban or a rural setting?

- Predominantly urban
- Predominantly rural
- Half urban and half rural

16. Do you work in a public or a private setting?

- Predominantly public

- Predominantly private
- Half public and half private

17. Is your practice predominantly in

- Anaesthesia
- Intensive care
- Pain medicine
- Other (Please specify)

Thank you for completing this survey.
