# Supplementary material for: Anaesthetists’ attitudes towards attending the funerals of their patients: A cross-sectional study among Australian and New Zealand anaesthetists
Source: PLoS One. 2020 Nov 5;15(11):e0239996. doi: 10.1371/journal.pone.0239996 (PMC7643987; doi:10.1371/journal.pone.0239996)
Supplement: S2 Appendix — (PDF) [file pone.0239996.s002.pdf]

Dear Director of Anaesthesia

All anaesthesia Fellows registered with ANZCA are invited to participate in a survey about the current attitudes of anaesthetists' attendance at the funeral of patients they cared for. We would appreciate this email to be forwarded to all consultant anaesthetists in your Department. The aim of our study is to describe the attitudes of anaesthetists attending the funeral of a patient that they care for.

Participation in this survey is voluntary. All information is anonymous and confidential. No internet provider addresses are being collected. This survey consists of 11 questions and is estimated to take approximately 5 minutes to complete.

Our survey is being sent to a randomised selection of ANZCA Fellows. It has ethics approval from the Austin Health Low Risk Ethics Panel (number: LNR/17/Austin/422).

Participant Information Sheet

For more information about the project, click, or copy and paste into your browser: https://www.dropbox.com/s/egveo8pa2j0v80d/20170823%20-%20Funeral%20SURVEY%20PICF_Ver_1.pdf?dl=0

Accessing the survey

Please click on the link below to participate in the survey, or copy and paste into your browser:

https://www.surveymonkey.com/r/JPZJC7R

Thank you in advance for your valuable contribution to this research.

A/Professor Laurence Weinberg

Contact: A/Professor Laurence Weinberg: laurence.weinberg@austin.org.au

---
