# Supplementary material for: Anaesthetists’ attitudes towards attending the funerals of their patients: A cross-sectional study among Australian and New Zealand anaesthetists
Source: PLoS One. 2020 Nov 5;15(11):e0239996. doi: 10.1371/journal.pone.0239996 (PMC7643987; doi:10.1371/journal.pone.0239996)
Supplement: S3 Appendix — (PDF) [file pone.0239996.s003.pdf]

Quality Assurance Participation Information and Consent Form

Participant Information and Consent Form

Version 1 – Dated 11/08/2017

Site – Austin Health

Full project title: Attitudes of anaesthetists attending the funeral of patients they care for: a cross-sectional survey amongst Australian anaesthetists

Principal Researcher: Dr Laurence Weinberg

Associate Researcher: Dr Kwangtaek Kim

This Participant Information Consent Form is 2 pages long. Please make sure you have all the pages.

1. Your Consent

You are invited to take part in this research project. Participation is voluntary and you may decline if you wish. However, due to anonymous data collection, we will be unable to remove your responses should you wish to revoke your consent after completion of the survey. If you choose not to participate in this study, this will not impact upon your employment with your current health organisation or your standing with ANZCA.

Please read this information and ask questions if you need more information. Once you understand what the project is about and if you agree to take part in it, please complete the survey and your consent will be taken as given by your participation

2. Purpose

You are invited to participate in this research project as a fellow of ANZCA. We are investigating attitudes of anaesthetists when they attend the funeral of a patient they care for, and what they perceive as benefits of, and barriers to the attendance.

3. Procedures

Participation in this project will involve completion of the following survey, taking approximately 5 minutes.

4. Privacy, Confidentiality and Disclosure of Information

The information collected via this survey will be entirely confidential and non-identifiable. Internet Protocol (IP)tracking will only be employed by the Survey

Monkey system to ensure single submissions only and this information will not be accessible by the researchers.

5. Results of Project

Results of this project will be presented at Austin Health Anaesthetics Department. We will also be aiming to publish these results in a peer reviewed journal.

7. Contacts

The person you may need to contact will depend on the nature of your query. If you want further information concerning this project or if you have any problems which may be related to your involvement in the project, you can contact the researchers at:

Name: Dr Laurence Weinberg
Position: Principal Researcher
Telephone: (03) 94965429
Email: Laurence.WEINBERG@austin.org.au

.
